# Experience with Atmospheric Plates

**Published:** 1859-09

**Authors:** A. Berry


### EXPERIENCE WITH ATMOSPHERIC PLATES FOR PARTIAL SETS OF TEETH.

BY A. BERRY, D. D. S.

SINCE a large proportion—probably seven-eighths of the teeth to which clasps are applied become carious in a few years as the result of clasping, it is very important to avoid this mode of sustaining teeth when practicable.

Dr. Thompson, in the American Journal of Dental Science, April, 1859, describes his mode of making clasps, which is very good as securing the closest fitting of the clasps to the teeth, and adds, "We conceive that a tooth can not be injured when a clasp is thus formed—the adaptation being so perfect as to exclude all foreign matter from penetrating between the tooth and the clasp; it is, in fact, almost impervious to the fluids of the mouth."

Should any one do what Dr. T. has "*almost*" done, the clasp, if it rested on the gums, as often occurs, would cause caries sooner than if it did not fit closely.

To be sure there are cases, rarely met with probably, in which owing to defective form of the maxillary parts, small atmospheric plates can not be retained so firmly as to satisfy the patient. It is sometimes necessary to remove these plates from the mouth at meals, if the adjoining teeth must be used in mastication. But occasionally they are held so well as to be used in dividing the food.

Some eleven years ago—not having my dental journals at hand explicit dates can not be given—a description of Dr. Cleaveland's air-chamber was published in the Am. Journal of Dental Science. Having a patient needing the incisors inserted, with no teeth suitable for clasping, I resolved at once to see what could be done with the improvement, and made a piece which proved successful. During the two or three years ensuing I constructed several of these plates, and was highly pleased with the results. About this period Dr. Dwinelle's description of his valve for air-chambers, held in situ by a delicate spring in the chamber, was published. Believing that with the valve the air could be exhausted more completely than otherwise, I made two pieces with it, a nice troublesome job, with the success of which I was highly delighted, until after a few months one of my patients broke the spring and stopped the valve opening, *when the plate was retained in the mouth as firmly as ever.*

Soon after this time I adopted the chamber known as Gilbert's, and continue to use it.

Although a perfect impression may be obtained with wax, plaster is preferable. One of my friends who is very fortunate with such plates, removes the plaster from the mouth when it begins to "set," to avoid its holding about the remaining teeth. I prefer to build over spaces the teeth are to occupy on the sides of the wax holder with the plates of wax, then to fill the other part with plaster, and let it harden well before taking it from the mouth.

For raising a plaster cast for the chamber, which I like in most cases to have a little extending on the parts rising towards the alveolar portion, I place adhesive wax rolled to proper thickness with its edges nearly to right angles to the parts it rests on, and keep it in place by the solution of gum used for varnishing the cast.

Gold of standard fineness is best for the plates; but since the introduction of California gold it is well to melt the coin before using it to obviate considerably the difficulty of holes occurring in it from fitting the plates. With a small slightly blunt instrument the plate should be driven down closely as possible around the border of the chamber. If an opening is made in the plate it may be known by the whistling produced by pressing it home in the mouth—a sound quite different from that made by air passing out around the borders of the piece. To find the exact locality of such holes is sometimes difficult; but I have succeeded, even when they were very small, by holding the plate close to one eye before a strong light, passing a finely pointed instrument about until it comes over the opening, when a mark is made showing where to flow the solder.

When the rugæ on the posterior part of the alveolar ridge are prominent they often prevent the pieces from being held so firmly as they otherwise would be. The plate may be cut away to one-fourth of an inch in width over the median line, where if there are any rugæ they are commonly small, and another thickness of plate soldered on it. If the piece be for more than one tooth, a narrow strong plate under and a little projecting behind the teeth will answer. I usually do not bring it over in front of the alveolar ridge, but set the teeth close on the gum, for which, teeth with short gums made a few years ago by Jones, White & Co., are admirably adapted.

To prevent the plates from springing I *encase* them in sand and plaster deeply, to within half an inch of the teeth on pomice stone, then place the same materials slightly under and in front of the teeth, with a strip of platinum one-third inch wide in it to render it firmer. The soldering may then usually be done by confining the flame to the part about the teeth so as not to cause the plates to warp. But if the plates spring at all they may be fully restored by cutting away the male cast so that the teeth shall not touch it, sawing away the female cast to avoid the teeth, and striking them up again.

It is advantageous to make the female cast to be used in striking the last time, of some alloy melting at a much lower temperature than lead, to secure a closer fit of plate, by avoiding the large amount of contraction of lead in cooling.

Patients in most instances readily acquire the tact of exhausting the air from the chamber; but it is sometimes gained with difficulty—and I think in one or two cases my plates were not retained in the mouth from the air not being at all drawn from the cavity.

It is best when the alveolar ridge is low to have the plate worn a day or so before arranging the teeth on it, to avoid the danger of it coming forward in gaining its final position, and throwing the teeth out of line.

Neither teeth nor plate should be allowed, when in place, to touch the natural teeth, to afford free movement of the piece in accommodating itself to the parts as the air is withdrawn from the cavity.

During the last five years I have inserted about nine-tenths of my partial sets with the air-chamber; and in my humble opinion the air-chamber plate should be used much more extensively than it is by the profession generally.

July 15, 1859.

